# Perceived risk of diabetes seriously underestimates actual diabetes risk: The KORA FF4 study

**DOI:** 10.1371/journal.pone.0171152

**Published:** 2017-01-31

**Authors:** Bernd Kowall, Wolfgang Rathmann, Andreas Stang, Brenda Bongaerts, Oliver Kuss, Christian Herder, Michael Roden, Anne Quante, Rolf Holle, Cornelia Huth, Annette Peters, Christa Meisinger

**Affiliations:** 1 Center of Clinical Epidemiology, Institute for Medical Informatics, Biometry and Epidemiology, Medical Faculty, University Duisburg-Essen, Essen, Germany; 2 German Diabetes Center, Institute of Biometrics and Epidemiology, Düsseldorf, Germany; 3 German Center for Diabetes Research (DZD), München-Neuherberg, Germany; 4 School of Public Health, Department of Epidemiology Boston University, Talbot Building, Boston, Massachusetts, United States of America; 5 Institute for Clinical Diabetology, German Diabetes Center, Leibniz Center for Diabetes Research at Heinrich Heine University Düsseldorf, Düsseldorf, Germany; 6 Department of Endocrinology and Diabetology, Medical Faculty, Heinrich-Heine University, Düsseldorf, Germany; 7 Institute of Medical Informatics, Biometry and Epidemiology, Chair of Genetic Epidemiology, Ludwig-Maximilians-Universität, Munich, Germany; 8 Institute of Genetic Epidemiology, Helmholtz Zentrum München - German Research Center for Environmental Health, Neuherberg, Germany; 9 Institute of Health Economics and Health Care Management, Helmholtz Zentrum München, German Research Center for Environmental Health (GmbH), Neuherberg, Germany; 10 Institute of Epidemiology II, Helmholtz Zentrum München, German Research Center for Environmental Health (GmbH), Neuherberg, Germany; Baylor College of Medicine, UNITED STATES

## Abstract

**Objective:**

Early detection of diabetes and prediabetic states is beneficial for patients, but may be delayed by patients´ being overly optimistic about their own health. Therefore, we assessed how persons without known diabetes perceive their risk of having or developing diabetes, and we identified factors associated with perception of diabetes risk.

**Research design and methods:**

1,953 participants without previously known diabetes from the population-based, German KORA FF4 Study (59.1 years, 47.8% men) had an oral glucose tolerance test. They estimated their probability of having undiagnosed diabetes mellitus (UDM) on a six category scale, and assessed whether they were at risk of developing diabetes in the future. We cross-tabulated glycemic status with risk perception, and fitted robust Poisson regression models to identify determinants of diabetes risk perception.

**Results:**

74% (95% CI: 65–82) of persons with UDM believed that their probability of having undetected diabetes was low or very low. 72% (95% CI: 69–75) of persons with prediabetes believed that they were not at risk of developing diabetes. In people with prediabetes, seeing oneself at risk of diabetes was associated with self-rated poor general health (prevalence ratio (PR) = 3.1 (95% CI: 1.4–6.8), parental diabetes (PR = 2.6, 1.9–3.4), high educational level (PR = 1.9 (1.4–2.5)), lower age (PR = 0.7, 0.6–0.8, per 1 standard deviation increase), female sex (PR = 1.2, 0.9–1.5) and obesity (PR = 1.5, 1.2–2.0).

**Conclusions:**

People with undiagnosed diabetes or prediabetes considerably underestimate their probability of having or developing diabetes. Contrary to associations with actual diabetes risk, perceived diabetes risk was lower in men, lower educated and older persons.

## Introduction

According to the International Diabetes Federation (IDF), almost half of the people with diabetes worldwide are unaware of having the disease, and even in high-income countries, about one in three diabetes cases is not diagnosed [1,2]. In the USA, 28% of diabetes cases are undiagnosed [3]. In DEGS1, a recent population-based German survey, 22% of persons with HbA1c ≥ 6.5% were unaware of their disease [4].

Persons with undiagnosed diabetes mellitus (UDM) have a more than twofold risk of mortality compared to persons with normal glucose tolerance (NGT) [5,6]; many of them also have undiagnosed diabetes complications like retinopathy and chronic kidney disease [7,8]. Colagiuri and Davies have summarized several reasons why persons benefit from early detection of diabetes [9]. In particular, early detection of diabetes leading to lifestyle changes and medical treatment can reduce cardiovascular morbidity and mortality as well as prevalence and severity of retinopathy [10,11].

Likewise, prediabetes—a state of elevated levels of blood glucose that still are below the threshold for type 2 diabetes—is highly prevalent in Western countries but people are often unaware of this condition [12]. People with prediabetes have an increased risk of developing diabetes with the annual progression risk being about 5–10% depending on population characteristics. People with a combination of impaired fasting glucose (IFG) and impaired glucose tolerance (IGT) have an even higher risk of progressing to diabetes [12,13]. Nevertheless, progression from prediabetes to diabetes can often be prevented by even moderate lifestyle intervention or by metformin [14–17].

Thus, early detection of diabetes and prediabetes is beneficial for patients, but may be delayed by patients´ being overly optimistic about their own health. Therefore, it is important to address how persons with UDM or prediabetes perceive their diabetes risk. So far, perception of diabetes risk has been compared to measured glycemic status only in the Dutch Hoorn Study [18,19]. The aim of our study was (i) to assess how people with UDM estimate their likeliness of having diabetes, (ii) to assess how people with prediabetes perceive their diabetes risk, (iii) to identify factors associated with perceived diabetes risks, and (iv) to analyze whether simple non-invasive diabetes risk scores are superior to perceived risk for the identification of UDM.

## Research design and methods

### Study population

The KORA FF4 study is the second follow-up of the KORA S4 study, a population-based health survey conducted in the city of Augsburg and two surrounding counties between 1999 and 2001. A total sample of 6640 subjects was randomly drawn from the target population consisting of all German residents of the region aged 25 to 74 years.

Of all 4261 participants of the S4 baseline study, 2279 also participated in the 14-year follow-up FF4 study. The study was conducted from June 2013 to September 2014, and participants had to be physically present in the study center. Persons were considered ineligible for FF4 if they had died in the meantime (n = 455, 10.7%), lived too far outside the study region or were completely lost to follow-up (n = 296, 6.9%), or had demanded deletion of their address data (n = 191, 4.5%). Of the remaining 3319 eligible persons, 157 could not be contacted, 504 were unable to come because they were too ill or had no time, and 379 were not willing to participate in this follow-up, giving a response of 68.7%.

The KORA FF4 study is not focused on diabetes, but it is a broader study of health characteristics, health related behavior and health related perceptions. It includes face-to-face interviews and a thorough physical examination including among others anthropometric measurements and comprehensive laboratory tests.

The investigations were carried out in accordance with the Declaration of Helsinki, including written informed consent of all participants. All study methods were approved by the ethics committee of the Bavarian Chamber of Physicians, Munich.

### Ascertainment of type 2 diabetes and prediabetes

Previously known diabetes was defined as self-report and validated by questioning the responsible physician, or as current use of glucose-lowering agents. All participants without known diabetes were to receive a standard oral glucose tolerance test (OGTT), carried out in the morning (7:00 am to 11:00 am). Participants were asked to fast for 10 h overnight, to avoid heavy physical activity on the day before examination and to refrain from smoking before and during the test. Exclusion criteria for the OGTT were: (i) consumption of foods or drinks containing calories within 8 h before the fasting blood draw; (ii) medical contraindications such as gastrointestinal disease, fructose intolerance, currant allergy, weakness, risk of hypoglycemia, or pregnancy. Fasting venous blood was sampled for glucose determination after which 75 g of anhydrous glucose was given (Dextro OGT, Boehringer Mannheim, Germany, containing currant extract). The mean ± standard deviation duration for the 2-h glucose determination among all KORA OGTT participants was 120±1 min.

Previously undiagnosed diabetes (≥ 7.0 mmol/l fasting or ≥ 11.1 mmol/l 2-h post glucose load), impaired fasting glucose (IFG: fasting glucose 5.6–6.9 mmol/l) and impaired glucose tolerance (IGT: 2-h glucose 7.8–11.0 mmol/l) were defined according to the 2003 ADA diagnostic criteria [20]. Prediabetes included isolated IFG, isolated IGT, and combined IFG and IGT. In the following, we will use UDM as an abbreviation for previously undiagnosed diabetes, which is synonymous with screen-detected diabetes.

### Perception of diabetes risk

Persons did not know their outcome of the OGTT at the time of the interview.

Participants who did not give a self-report of physician diagnosed diabetes were asked the following three questions: (1) “How do you estimate the risk that you have diabetes at the present moment but do not know about it: negligible—very low—low—neither low nor high—high—very high.” (2) “Do you believe that you are at risk of developing diabetes in the next years: Yes—No—I don´t know.” (3) “How serious a disease is diabetes in your view? I consider diabetes as: not a serious disease—a moderately serious disease—a serious disease—a very serious disease—I don´t know.”

### Measurement of covariates

Height, weight, waist circumference, systolic and diastolic blood pressure were measured based on standard protocols as described elsewhere [21]. Trained medical interviewers gathered information on sociodemographic variables, physical activity, alcohol consumption, smoking and parental history of diabetes.

Body mass index (BMI) was calculated as weight in kilograms divided by height squared (in square meters). Hypertension was defined as systolic blood pressure of 140 mm Hg or higher, diastolic blood pressure of 90 mm Hg or higher, or use of antihypertensive medication given that the subjects were aware of being hypertensive. Subjective health was measured on a four-categorical scale (very good / good / less good / poor), but for regression analyses, the two latter categories were combined. Persons were defined as physically inactive if they exercised regularly less than at least one hour per week. Parental diabetes was defined as either paternal or maternal diabetes, or both. School leaving certificates were categorized as low, intermediate and high according to the three school types at secondary level in the three-tiered German school system.

Serum glucose was analyzed using a hexokinase method (GLUFlex, Dade Behring, Deerfield, IL, USA). Glucose, HDL- and LDL-cholesterol as well as triglyceride levels were measured in fresh fasting serum by enzymatic, colorimetric methods using GLU, LDLC, HDLC, and TRIG Flex assays on a Dimension Vista 1500 instrument (Siemens Healthcare Diagnostics Inc., Newark, USA) or GLUC3, LDL_C, HDLC3, and TRIGL assays on a Cobas c701/702 instrument (Roche Diagnostics GmbH, Mannheim, Germany). The measurement instrument and assays changed from Siemens to Roche during the study. Calibration formulas were developed using 122 KORA FF4 samples which were measured with both methods during the time of the change. The Siemens measurement results were calibrated to the Roche measurements using the following formulas [all units in mg/dl]: HDL_Cholesterol_Roche = 2.40 + HDL_Cholesterol_Siemens * 1.12; LDL_Cholesterol_Roche = antilog (-0.13328 + log LDL_Cholesterol_Siemens * 1.03051); Triglycerides_Roche = 4.97073 + Triglycerides_Siemens * 0.90732. No calibration was needed for the glucose assessment because the double measurements were very similar so that the intercept and the slope of the Passing-Bablok regression used for calibration were estimated to be zero and one, respectively. HbA1c was measured in hemolyzed whole blood using the cation-exchange high performance liquid chromatographic, photometric VARIANT II TURBO HbA1c Kit—2.0 assay on a VARIANT II TURBO Hemoglobin Testing System (Bio-Rad Laboratories Inc., Hercules, CA, USA).

### Statistical analyses

We present the perceived risk of having UDM at the time of the interview, and the perceived risk of developing diabetes over the next years, respectively, by categories of glucose status.

We fitted Poisson regression models with robust error variance to estimate prevalence ratios with 95% confidence intervals for the association between perceived risk of developing diabetes (yes versus no / don´t know) as the dependent variable and various potential determinants of risk perception as independent variables [22]. These analyses were confined to persons with prediabetes because these people actually have an increased risk of developing diabetes. We fitted separate models for each independent variable, and, in addition, a full model with all independent variables.

We compared the perceived risk of having UDM with two non-invasive diabetes risk scores (KORA [23], DESIR [24]) with regard to the identification of UDM. The KORA score comprises age, sex, BMI, parental diabetes, hypertension, and smoking (current, former, never); the DESIR comprises sex, waist circumference, parental diabetes, hypertension and smoking. To this purpose, we estimated the area under the receiver operating characteristic curve (AROC) from logistic regression models with self-assessed risk of UDM or one of the two non-invasive risk scores as the independent variable and presence of UDM as the dependent variable.

The analyses were carried out using SAS version 9.4.

## Results

Among the 2,279 participants, glycemic state could not be ascertained for 93 persons due to lack of an oral glucose tolerance test. Of the remaining 2,186 persons, 233 (10.7%) had previously known diabetes, 94 (4.3%) had UDM, 773 (35.4%) had prediabetes, and 1,086 (49.7%) had normal glucose tolerance (NGT). Persons with prediabetes or diabetes comprised more men than women compared to people with NGT (Table 1). Moreover, persons with diabetes had a less favorable metabolic profile (i.e., larger waist circumference, higher serum levels of triglycerides, a larger proportion of hypertension) than persons with prediabetes who in turn showed a less favorable metabolic profile than persons with NGT. The proportion of persons with less good or poor subjective health was higher in persons with diabetes than in persons with prediabetes or NGT (Table 1). Almost nine in ten persons without previously known diabetes perceived diabetes as a serious or very serious disease.

**Table 1:** Basic characteristics of the study group by categories of glucose regulation: The KORA FF4 study ^a^.

	Normal glucose tolerance	Prediabetes	Undiagnosed diabetes	Previously known diabetes
N	1,086	773	94	233
Age (years)	55.7 ± 11.3	62.8 ± 11.5	68.4 ± 10.6	69.7 ± 10.0
Sex (male) (%)	37.9	60.5	58.5	57.5
BMI (kg/m^2^)	26.1 ± 7.4	28.9 ± 4.7	31.3 ± 5.5	31.0 ± 5.2
Waist circumference (cm)	91.0 ± 12.7	101.0 ± 12.6	107.0 ± 13.5	107.3 ± 12.6
HDL cholesterol (mmol/l)	1.81 ± 0.49	1.60 ± 0.45	1.46 ± 0.42	1.51 ± 0.43
LDL cholesterol (mmol/l)	3.45 ± 0.88	3.61 ± 0.91	3.63 ± 1.04	3.13 ± 0.87
Triglycerides (mmol/l)	1.03 (0.77; 1.38)	1.33 (0.98; 1.82)	1.62 (1.25; 2.46)	1.43 (1.09; 2.08)
Hypertension (%)	23.5	47.4	63.8	76.7
Serum fasting glucose (mmol/l)	5.1 ± 0.3	5.8 ± 0.4	7.2 ± 1.8	7.9 ± 2.1
Serum 2-hour glucose (mmol/l)	5.2 ± 1.1	7.0 ± 1.8	12.5 ± 3.6	-
HbA1c [%]	5.3 ± 0.3	5.8 ± 0.4	6.2 ± 0.9	6.7 ± 1.1
HbA1c (mmol/mol)	34 ± 4	40 ± 4	44 ± 10	50 ± 12
Parental diabetes (%)				
Yes ^b^	27.9	34.4	40.4	47.2
No	63.6	55.0	42.6	39.1
Don’t know	8.5	10.6	17.0	13.7
Smoking (%)				
Current	17.6	15.0	7.5	8.6
Former	35.0	41.4	38.3	47.6
Never	47.4	43.6	54.3	43.8
Physical activity (inactive) (%) ^c^	35.1	44.2	66.0	60.9
School leaving certificate (%)				
Low	39.5	54.8	62.8	65.7
Intermediate	31.0	21.4	20.2	17.6
High	29.4	23.8	17.0	16.7
Medical check-up at least once (%)	56.1	51.9	47.3	50.9
Perception of diabetes as a serious or very serious disease (%)	90.5	86.9	75.5	-
Subjective health (%)				
Very good	17.1	8.4	5.3	6.9
Good	67.2	71.9	63.8	59.2
Less good	14.8	17.6	24.5	27.5
Poor	0.8	2.1	6.4	6.4

^a^ Mean ± standard deviation, median (first quartile, third quartile), or proportion (%)

^b^ Father, mother or both had diabetes

^c^ Persons were defined as physically inactive if they exercised regularly less than at least one hour per week

The proportion of persons who perceived their risk of having UDM at the time of the interview as “negligible”, “very low” or “low” was 87.1% (95% CI: 85.0–89.0) in NGT, 83.9% (81.2–86.4) in prediabetes, and 74.2% (64.5–82.0) in UDM (Table 2). The proportion of persons who perceived themselves at risk of developing diabetes in the following years ranged from 14.6% (95% CI: 12.6–16.8) in NGT to 20.6% (17.9–23.6) in prediabetes to 28.7% (20.5–38.6) in UDM (Table 3). No differences in risk perception were seen between persons with isolated IFG, isolated IGT, and persons with both IFG and IGT.

**Table 2:** Perceived risk of having undiagnosed diabetes by categories of glucose regulation: The KORA FF4 study.

“How do you estimate the risk that you have diabetes at the present moment but do not know about it?”	Normal glucose tolerance	Prediabetes	Undiagnosed diabetes
N	1,086	772	93
Negligible	162 (14.9%)	58 (7.5%)	8 (8.6%)
Very low	450 (41.4%)	294 (38.1%)	26 (28.0%)
Low	334 (30.8%)	296 (38.3%)	35 (37.6%)
Neither low nor high	123 (11.3%)	104 (13.5%)	15 (16.1%)
High	17 (1.6%)	19 (2.5%)	9 (9.7%)
Very high	0 (0%)	1 (0.1%)	0 (0%)

**Table 3:** Perceived risk of developing diabetes in the next years by categories of glucose regulation: The KORA FF4 study.

“Do you believe you are at risk of developing diabetes in the next years?”	NGT	i-IFG	i-IGT	IFG / IGT	UDM
N	1,086	487	102	184	94
Yes	158 (14.6%)	102 (20.9%)	20 (19.6%)	37 (20.1%)	27 (28.7%)
No	866 (79.7%)	351 (72.1%)	74 (72.6%)	132 (71.7%)	61 (64.9%)
I don´t know	62 (5.7%)	34 (7.0%)	8 (7.8%)	15 (8.2%)	6 (6.4%)

NGT: normal glucose tolerance; i-IGT: isolated impaired fasting glucose; i-IGT: isolated impaired glucose tolerance; IFG/IGT: impaired fasting glucose and impaired impaired glucose tolerance; UDM: undiagnosed diabetes mellitus

**Fig 1:**
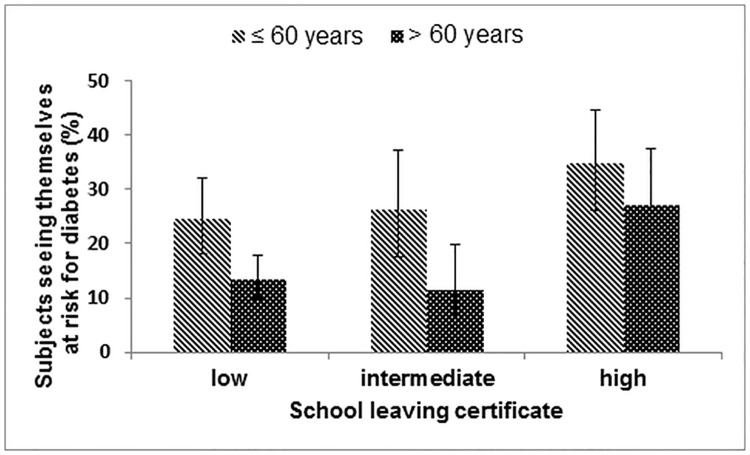
Subjective risk of diabetes by age and school leaving certificate.

In univariate regression models, perceiving oneself at risk of developing diabetes was associated with younger age, female sex, higher school education, obesity, self-rated poor general health, and parental diabetes (Table 4). Fig 1 shows that the proportion of better educated younger persons (age ≤ 60 years) with prediabetes, who perceived themselves at risk of developing diabetes was 35%, whereas this figure was only 13% in less well educated older persons (age > 60 years). Concentrations of fasting and 2-hour glucose, respectively, and HbA1c values were barely associated with perceived risk of developing diabetes. In a model including all potential determinants of diabetes risk perceptions, prevalence ratios were virtually the same except for obesity, and less good / poor subjective health.

**Table 4:** Associations between perceived risk of developing diabetes ^a^ and putative determinants of diabetes risk perception in persons with prediabetes. Results from robust poisson regression models (prevalence ratios (PR), 95% confidence intervals): The KORA FF4 study (N = 773).

Putative determinant	PR (95% CI) (separate models for each variable)	PR (95% CI) (fully adjusted model)
Age (per 1 SD) ^b^	0.7 (0.6; 0.8)	0.7 (0.6; 0.8)
Sex (reference: men)	1.2 (0.9; 1.6)	1.2 (0.9; 1.5)
BMI (per 1 SD) ^b^	1.4 (1.3; 1.5)	
Obesity (reference: no)	1.9 (1.4; 2.5)	1.5 (1.2; 2.0)
Physical activity (reference: active)	1.3 (1.0; 1.7)	1.2 (0.9; 1.6)
Parental diabetes		
Yes	2.6 (1.9; 3.5)	2.6 (1.9; 3.4)
Don’t know	1.3 (0.8; 2.3)	1.6 (1.0; 2.7)
No (ref.)	1	1
Subjective health		
Less good / poor	3.7 (1.7; 8.2)	3.1 (1.4; 6.8)
Good	2.0 (0.9; 4.3)	1.8 (0.9; 3.9)
Very good (ref.)	1	1
School leaving certificate		
High	1.8 (1.4; 2.5)	1.9 (1.4; 2.5)
Intermediate	1.1 (0.7; 1.6)	1.0 (0.7; 1.5)
Low (ref.)	1	1
Medical check-up at least once (ref.: no)	1.3 (1.0; 1.7)	1.3 (1.0; 1.6)
HbA1c (per 1 SD) ^b^	1.0 (0.9; 1.2)	1.0 (0.9; 1.2)
Fasting glucose (per 1 SD) ^b^	1.0 (0.9 1.2)	1.0 (0.9; 1.1)
2-hour glucose (per 1 SD) ^b^	1.0 (0.9; 1.2)	1.1 (0.9; 1.2)

PR: prevalence ratio; SD: standard deviation

^a^ Answer to “Do you believe you are at risk of developing diabetes in the next years?” (yes vs no / don’t know)

^b^ The standard deviations were 11.53 years for age, 4.66 kg/m^2^ for BMI, 0.34% for HbA1c, 0.406 mmol/l for fasting glucose, and 1.804 mmol/l for 2-hour glucose.

In the present study, the KORA and the DESIR score both discriminated better between persons with and without UDM than did the subjective risk assessment. AROCs were 0.80 (95% CI: 0.76; 0.84) (KORA), 0.73 (0.68; 0.77) (DESIR), and 0.60 (0.54; 0.66) (self-perceived diabetes risk). The differences of the AROCs were 0.20 (95% CI: 0.13; 0.27) (KORA versus self-perceived risk), and 0.13 (0.06; 0.19) (DESIR versus self-perceived risk).

## Conclusions

The present study shows that three out of four persons with UDM believed that the probability of having undetected diabetes was low or very low. In persons with prediabetes, more than 70% believed that they were not at risk of developing diabetes in the next years. People with prediabetes were more inclined to perceive themselves at risk of diabetes if their self-rated general health was poor, their mother or father had diabetes, they were obese, they were female, their educational level was high, and if they were younger. Comparing perceived risk of having UDM with non-invasive diabetes risk scores, the latter were better at identifying people with UDM.

Underestimating one´s diabetes risk indicates an optimistic bias concerning negative health outcomes. This bias is a known phenomenon. Weinstein suggested potential explanations for optimistic bias about susceptibility to health problems [25]: Unrealistic optimism is more likely when people lack previous experience with a disease, and when they perceive their disease risk as controllable. Lack of previous experience and perceived controllability is likely to be smaller in persons with a family history of diabetes, and, therefore, larger perceived risk in people whose parents had diabetes is plausible. A recent study on 660 college students demonstrated optimistic bias: 68% believed that other students had a higher diabetes risk than they had, but only 23% believed they were at a higher risk than other students [26].

In the present study, four associations with perceived diabetes risk were contradictory to established associations with actual diabetes risk. First, although diabetes risk increases with age, in persons with prediabetes, the association between age and perceived risk of developing diabetes was negative. This is in line with results by Adriaanse et al. who suggested as an explanation that older people perceive a lower diabetes risk because of their shorter life expectancy [18,19]. Second, among persons with prediabetes, those with high school leaving certificates more often believed that they were at risk of developing diabetes. This may be explained by higher health literacy in persons with a higher educational level [27]. Thus, differences in risk perception may add to the well-known explanations of the fact that the higher educated are at a lower diabetes risk [28]. Third, females with prediabetes perceived themselves at risk for diabetes more often than men, although, like in the present study, more men than women develop diabetes. Fourth, persons with prediabetes did not perceive themselves at higher risk of diabetes if they had higher levels of HbA1c or if they had both IFG and IGT. Presumably, persons with prediabetes have little information about their glycemic state.

In our study, non-invasive diabetes risk scores were better than subjective risk assessment at identifying UDM. One might argue that the KORA score was developed in earlier phases of the KORA study (albeit with partially different participants), and, thus, performs particularly well in the KORA FF4 study. However, this is not true for the DESIR score, which was developed in a completely different study cohort. The DESIR score includes only very few parameters (sex, hypertension, smoking, waist circumference, parental diabetes), and has good discriminatory power [24].

### Comparison with other studies

The present results were in line with results from the Dutch Hoorn Study [18,19]. Adriaanse et al. reported that among persons with UDM, only 28.3% perceived their likeliness of having diabetes to be at least 10% [18], and among persons with high risk of diabetes (predicted from a symptom risk questionnaire), the median perceived likeliness of having diabetes was 10.8% [19]. Again, perceived risk did not fully reflect the actual risk profiles. For BMI, there was barely any association with perceived risk of diabetes in the Dutch study [19].

For 569 British persons free of diabetes, Godino et al. found that perceived diabetes risk increased with higher values of the Framingham Diabetes Risk Score (FDRS), higher HbA1c, higher self-reported weight and poorer self-rated health [29]. In 150 primary care patients without diabetes from Massachusetts (USA), high perceived risk was associated with a less favorable metabolic risk profile and higher FDRS values, but not with greater intentions to change lifestyle [30]. In the two latter studies, the authors did not assess perceived diabetes risk in persons with validated prediabetes or diabetes. Therefore, the discrepancy between perceived diabetes risk and objective glycemic status (presence of UDM or prediabetes) is more evident in the present study and in the Dutch Hoorn Study.

### Public health relevance of the results

The Protection Motivation Theory (PMT) is widely applied to understand under which conditions people change their behavior to protect themselves from health risks [31]. According to this theory, four conditions must be fulfilled so that people build an intention to change their health related behavior: they must perceive the disease as severe; they must perceive themselves as vulnerable, i.e., to have a high perceived disease risk; they must consider the recommended health behavior as effective to reduce the risk, and, finally, they must perceive themselves as able to perform the respective behavior.

Applying the PMT to the case of diabetes, the first condition (disease severity) can be seen as fulfilled: in the present, but also in other studies, a large majority of persons considered diabetes as a serious disease [19,29]. However, responding that diabetes is a serious condition may be the socially desirable answer in a health survey. The other conditions for behavior change are less well fulfilled. In the present study, the perceived diabetes risk (i.e., vulnerability) indicates an underestimate of the actual diabetes risk. Moreover, as shown by Hivert et al. [30], higher perceived diabetes risk does often not accord with greater intentions of behavior change—this indicates that people may not have an accurate knowledge which lifestyle changes are appropriate to reduce their risk [32], or they do not expect that behavior change can reduce diabetes risk (i.e., low response efficacy), or they do not believe in their ability to execute the recommended behavior changes (i.e., low self-efficacy).

For a more appropriate perception of diabetes risk and to improve response efficacy, non-invasive diabetes risk scores can be used to identify subjects with high diabetes risk [33]. Moreover, as shown in the present study, they may be suitable for detection of UDM. Such scores can be filled in in a few minutes without assistance from healthcare professionals, and, ideally, results should be combined with recommendations for lifestyle changes and, if necessary, for contacting doctors.

Our results showed that people with low and intermediate education strongly underestimate their risk of diabetes and may qualify as target groups for detection of UDM and prediabetes. However, conclusions are not straightforward for age: On the one hand, the older are prone to an optimistic bias with regard to their diabetes risk; on the other hand, the long-run consequences of diabetes are much more severe in the young—life-expectancy of fifty-year old people is about six years lower if they have a diagnosis of diabetes [34].

### Strengths and limitations

A strength of our study was the large, population-based sample with thorough phenotyping of participants. Moreover, we compared perceived risk with measurements of glucose concentrations including an OGTT.

Our study has several limitations. During the two earlier waves of the KORA study (KORA S4 and KORA F4 study), the participants had also undergone an oral glucose tolerance test. Subsequently, participants had been informed about their fasting and their 2-hour glucose levels (mg/dl) and their HbA1c value, but they had not been given any diagnosis. Instead, those with prediabetes or newly detected diabetes were advised to show their results to their doctor. We suppose that the information given to the participants in earlier waves of the KORA study might have influenced our results in the KORA FF4 study in two different directions. On the one hand, it might have given the participants a more realistic idea of their glycemic levels, and underestimation of diabetes risk would even have been stronger without that prior information. On the other hand, prior information of not having UDM or prediabetes might have suggested to the participants that they did not have diabetes or were not at risk of developing it. However, we assume that the prior information did not strongly affect risk perception in the third wave of the study: first, the two prior study waves had taken place 7 and 14 years earlier, respectively. Second, we guess that many participants did not pay much attention to the information about glucose levels and HbA1c: from a small subsample of the KORA S4 study, we know that only about 60% of those advised to show their results to the doctor actually did so.

To assess perceived risk, we used categories instead of percent scales from 0 to 100. This may lead to misclassification. However, many people have difficulties in understanding percentages [35], and we doubt whether most people actually perceive risks in terms of percentages. Moreover, there were many older and elderly people in our sample, and about half of the participants had a low school leaving certificate. We supposed that these people might have particular difficulty transforming subjective risk into probabilities; therefore, we used categorical measures of perceived risk. A limitation of our study is that we had no items on knowledge of diabetes risk factors. Therefore, we could not assess whether people with better knowledge of diabetes risk factors had a more adequate perceived risk of diabetes. Finally, we studied a German sample of European descent, results may therefore not be generalizable to people with other ethnic and/or cultural backgrounds.

To conclude, people with undiagnosed diabetes, and people with prediabetes strongly underestimate their probability of having or developing diabetes, respectively. Given the increasing risk of diabetes and the burden of the disease in Germany, and elsewhere, a combination of strategies to avoid misperceptions of diabetes risk may be considered. These might include: increasing knowledge about diabetes risk factors as part of secondary school education; making non-invasive risk scores easy to calculate and receive online and offline; routine measurements of glucose or HbA1c for obese people at hospitalization; and, increasing the awareness among general practitioners of how many persons have prediabetes or undiagnosed diabetes, and the likelihood that their patients underestimate their risks.
